# Genomic Prediction of Green Fraction Dynamics in Soybean Using Unmanned Aerial Vehicles Observations

**DOI:** 10.3389/fpls.2022.828864

**Published:** 2022-03-16

**Authors:** Yusuke Toda, Goshi Sasaki, Yoshihiro Ohmori, Yuji Yamasaki, Hirokazu Takahashi, Hideki Takanashi, Mai Tsuda, Hiromi Kajiya-Kanegae, Raul Lopez-Lozano, Hisashi Tsujimoto, Akito Kaga, Mikio Nakazono, Toru Fujiwara, Frederic Baret, Hiroyoshi Iwata

**Affiliations:** ^1^Graduate School of Agricultural and Life Sciences, The University of Tokyo, Tokyo, Japan; ^2^Arid Land Research Center, Tottori University, Tottori, Japan; ^3^Graduate School of Bioagricultural Sciences, Nagoya University, Nagoya, Japan; ^4^Tsukuba-Plant Innovation Research Center (T-PIRC), University of Tsukuba, Tsukuba, Japan; ^5^Research Center for Agricultural Information Technology, National Agriculture and Food Research Organization (NARO), Tokyo, Japan; ^6^Joint Research Unit of Mediterranean Environment and Modelling of Agroecosystems, National Research Institute for Agriculture, Food and Environment (INRAE), Avignon, France; ^7^Institute of Crop Science, National Agriculture and Food Research Organization (NARO), Tsukuba, Japan

**Keywords:** soybean, unmanned aerial vehicle, remote sensing, drought, green fraction, dynamics model, genomic prediction, genomic selection

## Abstract

With the widespread use of high-throughput phenotyping systems, growth process data are expected to become more easily available. By applying genomic prediction to growth data, it will be possible to predict the growth of untested genotypes. Predicting the growth process will be useful for crop breeding, as variability in the growth process has a significant impact on the management of plant cultivation. However, the integration of growth modeling and genomic prediction has yet to be studied in depth. In this study, we implemented new prediction models to propose a novel growth prediction scheme. Phenotype data of 198 soybean germplasm genotypes were acquired for 3 years in experimental fields in Tottori, Japan. The longitudinal changes in the green fractions were measured using UAV remote sensing. Then, a dynamic model was fitted to the green fraction to extract the dynamic characteristics of the green fraction as five parameters. Using the estimated growth parameters, we developed models for genomic prediction of the growth process and tested whether the inclusion of the dynamic model contributed to better prediction of growth. Our proposed models consist of two steps: first, predicting the parameters of the dynamics model with genomic prediction, and then substituting the predicted values for the parameters of the dynamics model. By evaluating the heritability of the growth parameters, the dynamic model was able to effectively extract genetic diversity in the growth characteristics of the green fraction. In addition, the proposed prediction model showed higher prediction accuracy than conventional genomic prediction models, especially when the future growth of the test population is a prediction target given the observed values in the first half of growth as training data. This indicates that our model was able to successfully combine information from the early growth period with phenotypic data from the training population for prediction. This prediction method could be applied to selection at an early growth stage in crop breeding, and could reduce the cost and time of field trials.

## Introduction

Genetic mechanisms of growth processes have become a crucial topic in plant breeding. The genetic dissection of the formation process of target traits of breeding such as yield quantity and quality will provide profound insights into its mechanism, which will lead to efficient selection of useful genotypes and rapid genetic improvement. This understanding is important in genomic selection (GS), where breeders skip field trials and select promising candidates based on the predicted breeding value provided by genomic prediction (GP) (Meuwissen et al., 2001). Most GP studies on crops have focused on traits at harvest, such as yield and quality (Krishnappa et al., 2021). If GP can predict genetic variation during the growth process, breeders can accurately determine the behavior of the genotypes obtained by GP and select the appropriate candidates. Further, growth prediction from the early period is likely to reduce the cost of field trials by shortening the period of observation.

However, the measurements required to derive data of growth trajectory are cost and labor intensive, representing a major bottleneck for genetic dissection, which requires the characterization of many genotypes. Due to the rapid development of sensing technologies in recent years, high-throughput phenotyping has become available for plant breeding, and the measurement of growth traits is becoming more practical. An accurate and detailed acquisition of growth processes through high-throughput measurements is expected to lead to improved genetic gains in plant breeding (Furbank and Tester, 2011; Cabrera-Bosquet et al., 2012; Araus and Cairns, 2014). For example, an automated phenotyping platform for the monitoring of three-dimensional plant growth in a greenhouse has enabled the genetic dissection of growth processes using a dynamic model (Campbell et al., 2018). In a field experiment, high-throughput phenotyping using unmanned aerial vehicles (UAVs) (Yang et al., 2017) and tractors (White et al., 2012) was used to measure plant growth. Among growth traits, the leaf area index (LAI) is often investigated because it is accessible from high-throughput phenotyping (Verger et al., 2014; Liu et al., 2017) while being sensitive to the environment, directly determining amount of light absorption, and thus affecting biomass production and yield. Until recently, however, these techniques were mainly used for crop management such as estimation of canopy state variables, soil properties and yield (Jin et al., 2018), and their applications to genetic dissection remain limited (Blancon et al., 2019).

Several methods have been proposed for the analysis of plant growth. One commonly used method involves fitting a growth model, such as Gompertz (Winsor, 1932) and logistic (Nelder, 1961), to the data and using the model parameters to quantify the dynamic pattern. This method can be applied to various types of dynamic measurements such as stem diameter of trees (Wu et al., 2004) and soybean canopy cover and height (Borra-Serrano et al., 2020). Several methods of quantitative genetics, such as quantitative trait loci analysis (Ma et al., 2002; Wu et al., 2002) and genome-wide association studies (Das et al., 2011; Crispim et al., 2015), have been applied to discover possible associations with growth model parameters. Growth models have also been used as a flexible tool to analyze various factors, such as the effect of selection in breeding (Piles et al., 2003) and the relationship among traits (Onogi et al., 2019). However, its application to GP has not been discussed in previous studies.

In this study, a method integrating a model of growth dynamics and GP was proposed and applied to investigate the growth of soybeans. We focused on the green fraction (GF) to model its dynamics. GF is defined as the fraction of green pixels in an image taken from the sky. This trait is a proxy for LAI and can be easily measured from UAV observations. The GF dynamics of soybean germplasm accessions were described using the parameters of a model consisting of logistic and exponential curves. Genetic variations in GF dynamics were quantified by decomposing the model parameters into genetic and residual effects using mixed models. Finally, the GP model is applied to predict the parameters of the GF dynamics model under a range of scenarios to illustrate the potential of the proposed method. A similar experiment was conducted in an earlier paper in which UAV-RS data was used as secondary traits to predict biomass (Toda et al., 2021a), while this study developed prediction models of growth curve itself.

## Materials and Methods

### Field Trials

**FIGURE 1:**
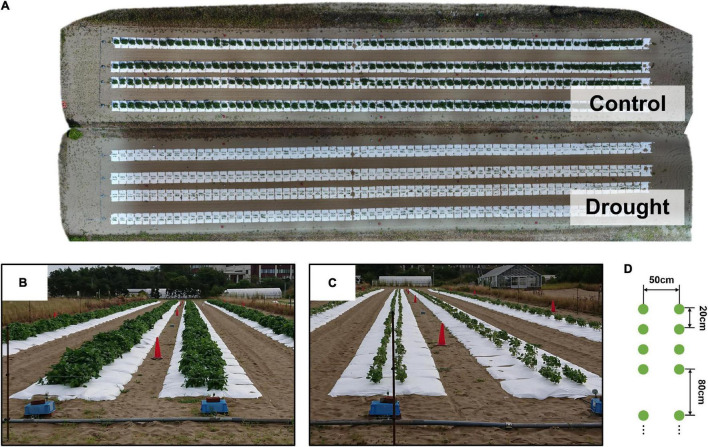
The field experiment. **(A)** An ortho-mosaic image of the field, obtained on August 25, 2018. The ortho-mosaic images were created for each treatment. **(B,C)** Ground level images of treatments C and D. **(D)** Planting pattern of plots made of 2 rows of 4 plants (green dots) and separated by 80 cm.

Soybean accessions registered in the National Agriculture and Food Research Organization Genebank^1^ were used. A total of 198 accessions, consisted of 96 Japanese accessions and 96 world accessions from mini core collection (Kaga et al., 2012) and 6 additional accessions. From 2017 to 2019, the field trial was conducted in an experimental field with sandy soil at the Arid Land Research Center, Tottori University (35°32′ N lat, 134°12′ E long, 14 m above sea level) (Supplementary Figure 1). A total of 198 accessions between 2018 and 2019 were used, with 186 out of 198 accessions used in 2017. Each plot consisted of four plants. The distances between two rows, two plots, and two individuals were 50, 80, and 20 cm, respectively (Figure 1D). Sowing was performed at the beginning of July, followed by thinning after 2 weeks (Supplementary Table 1). Fertilizer (15, 6.0, 20, 11, and 7.0 g m^–2^ of N, P, K, Mg, and Ca, respectively) was applied to the field before sowing.

Two watering treatment levels, control (C) and drought (D), were used to evaluate the genetic variations in the responses to water stress. White mulching sheets (Tyvek, Dupond, United States) were laid to prevent rainwater infiltration (Figure 1), and pipes were installed under the sheets to irrigate the field. Irrigation with irrigation rate 8.1 mm/h was applied for daily for 5 h (7:00–9:00, 12:00–14:00, 16:00–17:00), starting the day after the thinning in treatment C, while no water was brought in treatment D. In the following, an abbreviation for denoting a specific combination of the level of the treatment and the year of the experiment is used; for example, treatment C in 2017 is abbreviated to “2017-C.”

### Remote Sensing and Image Analysis

UAV flights started after thinning and were performed 16–35 times during the cultivation period. A consumer drone (DJI Phantom 4 Advanced, China) was used for image collection. Images consisted of RGB layers and 3,648 × 4,864 pixels, captured with an automated focus and white balance. The UAV flew 12–14 m above the ground and captured images every 2 s with an autofocus function. A single UAV flight took approximately 15 min and collected 500–600 images, which was repeated twice to cover the entire field.

Ortho-mosaic images were constructed using Pix4Dmapper (Pix4D, Switzerland). The images of individual plots were then segmented from the ortho-mosaic image based on the geolocation of their corners. The canopy regions of the images of the individual plots were segmented based on GRVI and hue values (GRVI < 0.05, 20 < Hue < 90). Finally, the GF of each plot was estimated as the ratio of the green pixels to the total number of pixels in the plot. The image analysis process was implemented in Python 3.7^2^ and library opencv (ver.4.1.0) and gdal (ver.3.2.2). For data in 2019, a similar procedure was used by Hiphen Inc.^3^ The analysis protocol was the same as previous research (Verger et al., 2014; Madec et al., 2017).

### Green Fraction Dynamics Modeling

**FIGURE 2:**
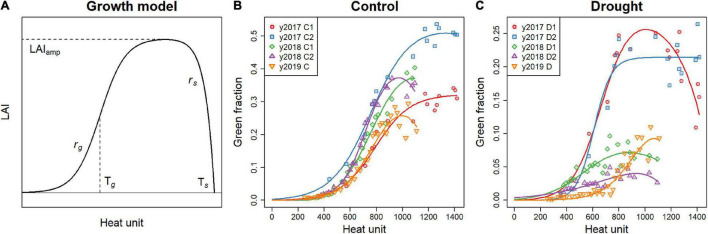
**(A)** An example of LAI dynamics described with the model (Equation 2). The parameters of the LAI dynamics model are displayed to visualize their roles. **(B,C)** GF dynamics of the genotype “Enrei,” with the model fitted to the data. Each plot is displayed separately for the control **(B)** and drought **(C)** treatments.

The GF derived from the UAV on day *d* day, GF*_*d*_*, was first converted into the corresponding leaf area index (LAI*_*d*_*), following (Soltani and Sinclair, 2012) the exponential model:


(1) $${\text{GF}}_{d}=1-\text{exp}\left(-k{\text{LAI}}_{d}\right)$$

where *k* = 0.5, and is the extinction coefficient commonly used for soybean. The model proposed by Koetz et al. (2005) to describe the dynamics of LAI was fitted to the time series of GF to estimate the growth pattern of each plot with five parameters (Figure 2):


(2) $${\text{LAI}}_{d}={\text{LAI}}_{\text{amp}}\left\{\frac{1}{1+\text{exp}\left(-r_{g}\left({\text{T}}_{d}-{\text{T}}_{g}\right)\right)}-\text{exp}\left(r_{s}\left({\text{T}}_{d}-{\text{T}}_{s}\right)\right)\right\}$$

The first term in the parenthesis represents logistic growth, and the second term represents exponential senescence, where T*_*d*_* is a growing degree day on day *d*. The growing degree day is a typical development scale corresponding to the cumulative daily mean temperature from sowing above the base temperature set to 8°C (Soltani and Sinclair, 2012). LAI_amp_ is the maximum value of the LAI reached, *r*_*g*_ is the maximum LAI growth rate, *r*_*s*_ is the senescence rate, T*_*g*_* is the growing degree day when the LAI growth rate is maximum, and T*_*s*_* is the growing degree day when LAI becomes zero. The five parameters, LAI_amp_, *r*_*g*_, *r*_*s*_, T*_*g*_*, and T*_*s*_*, were estimated for each plot. However, fitting the dynamics model separately for each plot was difficult because the GF in the drought treatment (D) was so small. Many of the GF growth data from the treatment D contained large noise, making it difficult to estimate the parameters, from data of each plot alone, especially the inflection point of the growth T*_*g*_*. Therefore, the estimation was conducted in two steps:

(1) First parameter estimates.

In this step, all of the parameters except LAI_amp_ were assumed to be genotype dependent and treatment independent, that is, parameters of each plot were the same for treatments C and D if their genotypes were the same. The optimal values of T*_*g*_* and T*_*s*_* were found with a grid search in the range 300 < T*_*g*_* < 1,200 and 1,400 < T*_*s*_* < 3,000 on seven points evenly distributed in the range. At the same time, the optimal values of the other parameters were estimated using the Nelder-Mead method. The cost function to be minimized in the Nelder-Mead method was computed as follows:


(3) $$\sum_{d}\sum_{i}\frac{{\left(y_{i,d}-{\hat{y}}_{i,d}\right)}^{2}}{{\bar{y}}_{d}},$$

where *y_*i*,d_* is the GF of plot *i* on day *d*, ${\hat{y}}_{i,d}$ is the estimated value of GF with the dynamics model, and ${\bar{y}}_{d}$ is the mean value of the GF on day *d*. The normalization by ${\bar{y}}_{d}$ in Equation 3 accounts for the measurement noise, which is roughly proportional to the mean value.

(2) Fine tuning the parameter estimates.

The parameter estimation was conducted independently for each plot. The optimal values obtained in the previous step for T*_*g*_* and T*_*s*_* were the center of the grid search with a narrower range (T*_*g*_*–200 < T*_*g*_* < T*_*g*_* + 200 and T*_*s*_*–400 < T*_*s*_* < T*_*s*_*+ 400). The other parameters were estimated using the Nelder-Mead method, using the estimated values in Step 1 as the initial values.

### Estimation of Genotypic Values

Genotypic values of the GF and LAI dynamic parameters were estimated for use in the GP. The following mixed model was fitted for each combination of a trait (GF or LAI dynamics parameter) and a treatment (C or D):


(4) y=μ1+Lβ+Ws+e

where **y** is a vector of the phenotypic values; *μ* is the mean; **β** is a vector of block effects representing differences between replications; **s** is a vector of genotypic values that follows N(**s** | **0**, σ_*s*_^2^**I**); σ_*s*_^2^ is the genotypic variance; **e** is a vector of residuals that follows N(**e** | 0, σ_*e*_^2^**I**); σ_*e*_^2^ is the residual variance; **1** is a vector in which all the elements are one; **I** is an identity matrix; and **L** and **W** are design matrices. The genotypic value (**g**) was calculated as follows:


(5) g=μ1+s

The R package lme4 (ver. 1.1–20) was used to solve Equation 4. For the GF, the genotypic value estimation was applied separately for each flight date.

### Genomic Relationship Matrix and Genetic Analysis

The whole-genome sequencing data of all 198 accessions were available and used to estimate the genomic relationship matrix (Kajiya-Kanegae et al., 2021). Only the biallelic sites in all accessions with a minor allele frequency (MAF) ≥ 0.025, missing rate < 0.05, and linkage disequilibrium < 0.95 were extracted, and the imputation of missing genotypes was applied. Genotyping data identified 425,858 SNPs. Genotypes for individual alleles were represented as -1 (homozygous for the reference allele), 1 (homozygous for the alternative allele), or 0 (heterozygous for the reference and alternative alleles). The genomic relationship matrix **G** was estimated as **G** = **XX**^T^ / *c*, where **X** is an *n* × *m* scaled marker genotype matrix (*n* and *m* are the numbers of lines and markers, respectively), and *c* is the normalization constant (Endelman and Jannink, 2012). Genetic heritability was estimated for all traits using the genomic best linear unbiased prediction (G-BLUP) model:


(6) g=m1+Zu+ε

where **g** is a vector of genotypic values estimated using Equations 4 and 5, *m* is the mean, **u** is a vector of random genetic effects that follows N(**u** | **0**, σ_*u*_^2^**G**), **ε** is a vector of residuals that follows N(**ε** | **0**, σ_ε_^2^**I**), σ_*u*_^2^ and σ_ε_^2^ are the genetic and residual variances, respectively, and **Z** is a design matrix. The R package rrBLUP (ver. 4.6) (Endelman, 2011) was used to solve Equation 6. After solving the mixed model, the genomic heritability was estimated as *h*^2^ = σ_*u*_^2^/(σ_*u*_^2^ + σ_ε_^2^).

### Prediction of Green Fraction Dynamics

We investigated three cross-validation schemes for the four different prediction models. The cross-validation schemes and prediction models are detailed as follows: The correlation coefficient between the genotypic values (**g**) and their predicted values (**u**) of the GF was used to evaluate the prediction accuracy.

#### Cross-Validation Schemes

Cross-validation was repeated three times for the combination of a cross-validation scheme and a prediction model.

*(1) Cross-validation of genotypes (CV1)*.

**FIGURE 3:**
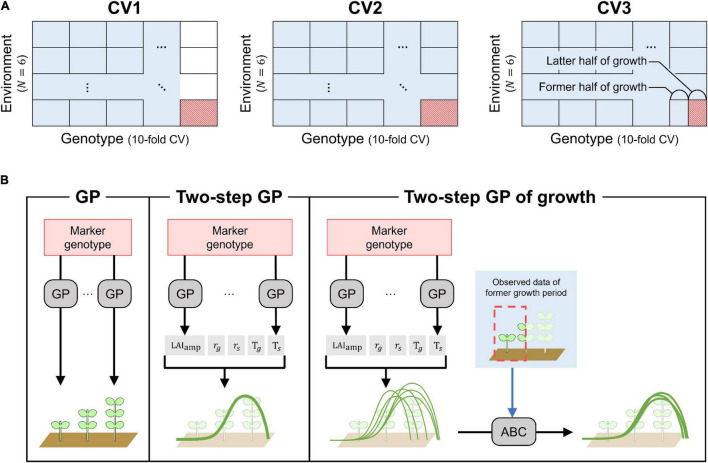
Cross-validation schemes and prediction models. **(A)** Cross-validation schemes (CV1, CV2, and CV3). Training and test data in cross-validation are expressed as blue and red cells, respectively. **(B)** Structures of prediction models (GP, TGP, and TGPG). Structures of the other prediction models with multivariate GP (MGP, TMGP, TMGPG) can be understood by replacing GPs in the figure to MGPs.

CV1 corresponded to the prediction of LAI dynamics for untested genotypes. Data from a subset of genotypes in any treatment or year were excluded from the training data (Figure 3A). The prediction model built using the training dataset was evaluated for the left-out genotypes. Ten-fold cross-validation was used to randomly select 19–20 left-out genotypes.

*(2) Cross-Validation over combination of genotype and environment (CV2)*.

The combination of a treatment and a year was considered as an environment: there were a total of six environments (two treatments × 3 years). Here, the predicted LAI dynamics were evaluated for genotypes and environments left out from the training dataset. A 10-fold cross-validation of genotypes and leave-one-environment-out cross-validation were applied simultaneously to get rid of data of test genotypes in one environment from training dataset (Figure 3A).

*(3) Cross-validation with a focus on the late growth period (CV3)*.

The growth cycle was split into early and late growth, with an equal number of observations for the two periods. CV3 was similar to CV2, but data of early growth period of test genotypes in a test environment was included in training data, and prediction of the LAI dynamics were evaluated over the late period (Figure 3A).

#### Prediction Models in Cross-Validation of Genotypes and Cross-Validation Over Combination of Genotype and Environment

In CV1 and CV2, four prediction models were compared: genomic prediction (GP), two-step GP (TGP), multivariate GP (MGP), and two-step multivariate GP (TMGP).

GP is the most standard model, expressed as shown in Equation 6, and applied to the GF on each day in the training data (Figure 3B). Then, the random genetic values g of the left-out genotypes were used as the predicted values.

The TGP consisted of two steps (Figure 3B). First, the same model as GP was applied to the LAI dynamics model parameters. Then, the GFs of the left-out genotypes on each day were calculated using the predicted parameters (Equations 1 and 2).

MGP is an extension of the GP, which simultaneously predicts several traits (Calus and Veerkamp, 2011; Jia and Jannink, 2012). The model is expected to enhance the accuracy of genomic prediction *via* genetic correlations among traits. This model can be expressed as follows:


(7) $$\begin{array}{c} \left(\begin{array}{c} {\text{g}}_{1}\\ \vdots \\ {\text{g}}_{J} \end{array}\right)=\left(\begin{array}{c} {\text{m}}_{1}1\\ \vdots \\ {\text{m}}_{J}1 \end{array}\right)+\left(\begin{array}{ccc} {\text{Z}}_{1} & \cdots & \text{O}\\ \vdots & \ddots & \vdots \\ \text{O} & \cdots & {\text{Z}}_{J} \end{array}\right)\left(\begin{array}{c} {\text{u}}_{1}\\ \vdots \\ {\text{u}}_{J} \end{array}\right)+\left(\begin{array}{c} \varepsilon_{1}\\ \vdots \\ \varepsilon_{J} \end{array}\right)\; \end{array}$$

where *J* is the number of variates in the model, **g***_*j*_*, **u***_*j*_*, and **ε***_*j*_* are vectors of genotypic values, random genetic effects, and residuals of variate *j*, respectively, and *m*_*j*_ is the mean of variate *j*. Assumptions for the random effects were included, in which **u**_all_ = (**u**_1_^T^, …, **u***_*J*_*^T^)^T^ follows N(**u**_all_ | **0**, **K**⊗ **G**) and **ε**_all_ = (**ε**_1_^T^, …, **ε***_*J*_*^T^)^T^ follows N(**ε**_all_ **| 0**, **R**⊗**I**). Here, **K** is a genomic variance-covariance matrix between the variates, and **R** is the residual variance-covariance matrix between the variates. The R package MTM (ver. 1.0.0) was used to solve Equation 7 based on the Markov chain Monte Carlo (MCMC) method.

TMGP consisted of two steps, that is, MGP of the LAI dynamics model parameters and the calculation of the GF using the predicted parameters.

MGP and TMGP were expected to improve the prediction accuracy compared to GP by exploiting phenotypic data from environments included only in the training dataset. However, since the GF was measured repeatedly in each environment, it was difficult to include all the phenotype data (152 measurements in total by adding up observation dates in all the environments). Thus, a strategy was applied where the training of prediction models was repeated for each observation date, and ten additional variates were selected from the whole data to support the prediction every time. In other words, the eleven variates included each time consisted of one target variate and ten supporting variates. The criterion for selecting supporting variates is based on heritability and correlation with the target variate. These two factors are essential for improving the prediction accuracy in MGP (Calus and Veerkamp, 2011) Top-10 observations of the following criterion were selected as supporting variables:


(8) $$\begin{array}{c} s\left(h^{2}\right)+s\left(\left|r\right|\right) \end{array}$$

where s(.) is a scaling function that makes the mean and variance of an input vector zero and one, respectively; *h*^2^ is the heritability; and *r* is the correlation coefficient with a target variate.

#### The Prediction Models in Cross-Validation With a Focus on the Late Growth Period

As in the other cross-validations, the performances of the four prediction models were compared in CV3. GP was the same as in CV2 because it only uses the data of the measurement day to be predicted for training. MGP was modified to better exploit the first half of the growth period used to train the model. Seven out of the ten supporting variates were selected using the selection criterion from Equation 8, the remaining three variates corresponded to the GF values for the latest three flights of the first growth period. The other two models with two-step structures, TGP and TMGP, were also modified to better exploit the training data for predicting the GF dynamics during the late growth period. They were called TGPG (TGP for growth) and TMGPG (TMGP for growth), respectively.

The TGPG included three steps (Figure 3B). The first two steps were the same as those of the TGP, where the LAI dynamics model parameters were predicted without using the data from the first half of the growth period. However, for TGPG, the distributions of the MCMC values of the LAI dynamics model parameters were used instead of the average value of the samples used in the TGP. As a result, 60,000 samples of the predicted GF dynamics were obtained for each genotype corresponding to the prior distribution when no GF measurements on the genotype were available. Then, the GF data from the first half of the growth period were exploited using the approximate Bayesian computation (ABC) method, and the 60 GF dynamics that minimize the Euclidian distance between the predicted GF dynamics and the actual GF observations were selected. Lastly, the mean values of the 60 samples were used as the predicted values. The modifications on TMGP to obtain TMGPG were the same as those applied to TPG to obtain TGPG.

## Results

### Dynamics of Green Fraction Derived From Unmanned Aerial Vehicles

**FIGURE 4:**
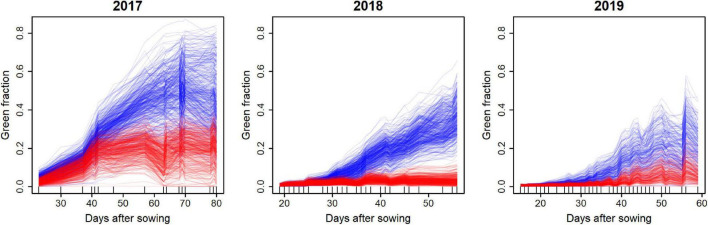
Dynamics of the GF as observed from the UAV. Treatments C and D are shown with blue and red lines, respectively, each line corresponding to a plot. The number of days after sowing is used as the x-axis. Small vertical bars on x-axis indicate dates of UAV-RS.

The UAV observations transformed into GF values show typical dynamics (growth, saturation, and senescence) of the several genotypes and environments investigated (Figure 4), showing large variations in the growth patterns. It is worth noting that the period covered by the fights was longer in 2017, with up to 80 days compared to 2018 and 2019, where the flights were stopped after 60 days. For each plot, the GF dynamics were relatively smooth, indicating a good temporal consistency of the GF values derived from the UAV observations. The ranking between genotypes is also generally consistent across growth development, which would indicate good chances to predict the late period from observations covering the early growth period. Drought treatment (D) always showed lower GF values than the control (C) treatment. However, the water stress experienced by treatment D varied across years, with 2018 being the most severe, and 2017 the mildest. The control treatment also showed differences between the 3 years: 2017 showed the best growth conditions, while 2019 showed the worst ones.

**FIGURE 5:**
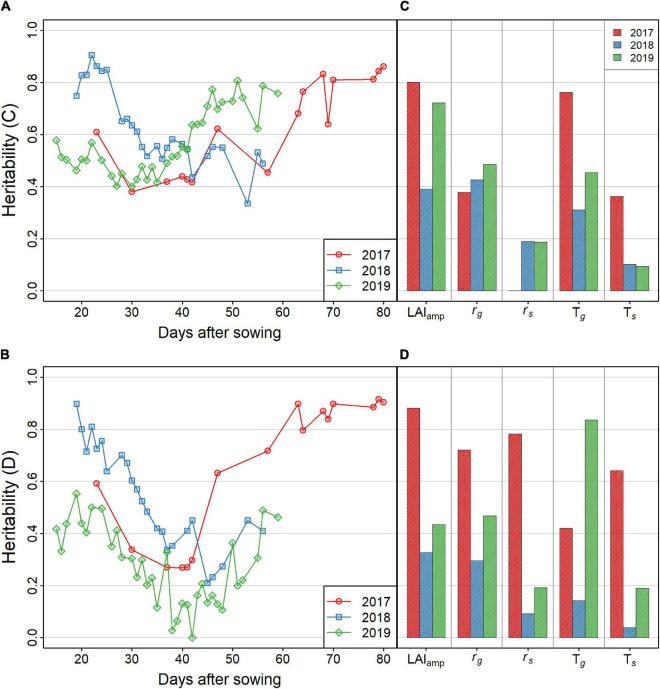
**(A,B)** Heritability of the GF. Results of treatments C and D are shown in **(A,B)**, respectively. Red, blue, and green lines indicate the values in 2017, 2018, and 2019, respectively. **(C,D)** Heritability of the LAI dynamics model parameters. Results of treatments C and D are shown in **(C,D)**, respectively.

The genomic heritability estimated for each year and treatment by fitting a mixed model to GF for each observation day showed a decrease until 40–50 days after sowing, and then increased with time (Figures 5A,B). However, some differences between years were observed, with a higher heritability in the early stages of 2018. The yearly patterns were also similar between the control (C) and drought (D) treatments, while the heritability in treatment C was systematically higher than that in treatment D, except in 2017 for the late UAV flights.

### Growth Parameter Estimation

The dynamics model fitted a wide range of GF growth patterns in both treatments and all years (Figure 2). The root mean squared errors (RMSE) of growth model fitting of GF on 25 days after sowing were 0.0060 and 0.0057 in treatment C and D, respectively. RMSE reached 0.022 and 0.016 in two treatments on 50 days after sowing, because growth of canopy increased the measurement noise of GF.

**FIGURE 6:**
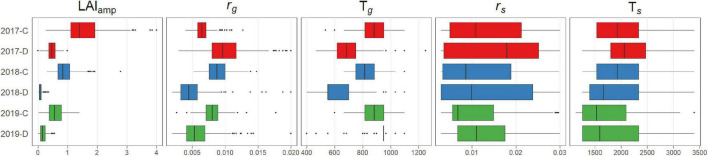
Boxplot of estimated growth parameters.

The distribution of the estimated growth parameters varied among years and treatments (Figure 6). Fundamentally, the parameters related to period of growth (*r*_*g*_, T*_*g*_*, and LAI_amp_) showed a tendency wherein the values of the parameters became smaller when the plants were subjected to drought stress. For the parameters related to period of senescence (*r*_*s*_, T*_*s*_*), the results were not reliable due to the lack of observation of senescence, except in 2017.

The genomic heritability of the growth parameters (Figures 5C,D) varied among treatments and years. The heritabilities of LAI_amp_, T*_*g*_*, and *r*_*g*_ were relatively high, reaching 0.8 in the highest cases. The other two parameters, *r*_*s*_ and T*_*s*_*, showed lower heritability, ranging between 0.1 and 0.5. These parameters characterize the late development of the canopy that was not well covered by UAV flights, except in 2017. This explains why heritability was highest in 2017. Particularly, heritability in 2017-D exceeded 0.4 with all values. On the other hand, heritability of all the parameters was lower than 0.4 in 2018-D when summer heat stress was severe. The heritability of the parameters of the model is generally lower than that of the GF for each UAV flight (Figure 5), except for LAI_amp_, which generally shows a higher heritability, except in 2018.

### Prediction of Growth Patterns

**FIGURE 7:**
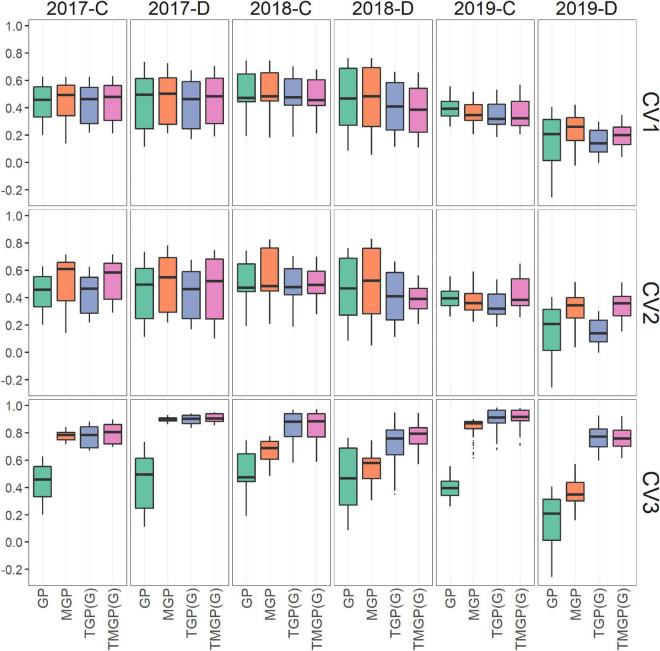
Comparison of accuracy of the prediction models. The correlation coefficients between genotypic values and their predicted values are plotted. The results of CV1, CV2, and CV3 are shown in three panels.

In CV1, the prediction accuracy of TGP and MGP was similar to that of GP (Figure 7). In 2019-D, a significant improvement in prediction accuracy in MGP (50.0% improvement in ratio of correlation coefficients of genotypic and predicted values) compared with GP was observed, where the accuracy of GP was very low in the latter half of the growth period (Supplementary Figure 2). The accuracy of MGP was higher (12.6%) than that of TGP. The accuracy of TMGP differed among environments; it was lower than that of GP when predicting the GF in 2018, while it was higher than the accuracy of MGP when predicting the GF in the latter half of the growth period in 2019.

The predicted values of GP and TGP in CV2 were equal to those in CV1 because they did not utilize data in environments other than their targets. Thus, for CV2, the focus will be on the accuracy of the MGP and TMGP. The accuracy of MGP was higher in CV2 than in CV1 (11.6%) and was significantly higher than that of TGP (25.6%) (Figure 7 and Supplementary Figure 3). The accuracy of TMGP was lower in CV2 than in CV1 in 2018, while it was higher in CV2 than in CV1 in the other years. Comparing MGP and TMGP, the accuracy of MGP was higher in 2018 and the former half of the growth period, while that of TMGP was higher in other environments.

In CV3, the prediction accuracy of TGPG and TMGPG was higher than that of the other models (24.5 and 27.1%, compared with MGP) in all the environments (Figure 7) over the entire growth period (Supplementary Figure 4). The correlation coefficients between the predicted values of TMGPG and the genotypic values were higher than 0.6 in most cases. The accuracy of MGP was higher than that of GP (71.7%), but lower than that of TGPG and TMGPG. As in CV2, the predicted values with GP and TGP in CV3 were the same as in CV1.

## Discussion

### Unmanned Aerial Vehicles-RS as a Tool to Evaluate Growth Patterns

This study showed that the UAV measurements of GF could be used to assess the genetic diversity of soybean growth patterns. A U-shaped longitudinal pattern was observed in the heritability of GF in all environments (Figures 5A,B). The U-shaped heritability patterns can be explained in three steps. In the early stage of growth, the GF seemed to be determined by factors regarding initial growth speed, such as radiation use efficiency, which results in high heritability of the GF. At approximately 25 days after sowing, several additional factors, such as growth phenology and plant structure, related to phenological development started to affect the GF, which decreased the heritability. Then, the saturation of the GF occurred around 45–60 days after sowing. During this period, confounding factors related to the differences in phenological development weakened, leading to increased heritability.

When we evaluate crop dynamics using remote sensing techniques, the LAI is often used as the main trait of interest. To apply remote sensing techniques to breeding populations, differences in plant styles should be carefully considered when deriving LAI from the images (Blancon et al., 2019). In this study, we focused on GF rather than LAI as the target trait to model its dynamics, due to the lack of data on plant style. Applying our method to LAI would offer the advantage of being closer to crop growth mechanisms, such as photosynthesis. Nevertheless, this study has shown that GF is a useful trait to describe the growth of soybean germplasm. In genomic prediction, the inclusion of canopy area, which is proportional to GF, has been reported to improve the prediction of biomass in soybean (Toda et al., 2021a). Thus, GF can be considered a useful index of genetic variation in plant growth.

### Fitting a Dynamics Model

The dynamics model was flexible enough to represent various growth patterns in different environments (Figure 2). By fitting the model, the GF time series is represented by five parameters. The distribution of growth parameters reflected the effects of drought stress on growth (Figure 6). The decrease in *r*_*g*_ and T*_*g*_* in treatment D indicates that the speed and duration of growth were strongly suppressed under drought stress. As a result, the maximum value of the plant canopy was significantly reduced, which was expressed in the reduction of LAI_amp_.

Genetic analysis of the parameters showed that the heritability of LAI_amp_ was the highest (Figures 5C,D). This result is related to the high heritability of GF in the later stages of growth. T*_*g*_*, which describes the stage-shift timing of the GF, also showed high heritability in 2017-C and 2019-D.

For the senescence stage, the heritability was low for T*_*s*_* and *r*_*s*_, except for 2017-D. Due to the long cultivation period in 2017 and early senescence in treatment D, model fitting of the senescence part was successful in that environment. The heritability of *r*_*s*_ was higher than T*_*s*_*, which means that the change in GF in the senescence stage was mainly determined by its speed, *r*_*s*_, rather than the timing of senescence, T*_*s*_*. The senescence pattern could be evaluated more precisely in 2017 and 2018 by extending the observation period.

Several useful results were obtained by applying the dynamics model, and some problems were found to be improved. Because of the high heritability of GF in the early stages, the growth speed, *r*_*g*_, was expected to be mainly determined by genetic factors. However, the heritability of *r*_*g*_ was low, except in 2017-D. The use of other dynamic models, such as the Gompertz (Winsor, 1932) curve, may improve the goodness of fit of a growth curve to the GF in the early growth stages. It was reported that the dynamics model that considers leaf appearance could explain the dynamics of green LAI (GLAI) (Blancon et al., 2019). Such structural models may also be candidates for alternative dynamic models.

Another possible improvement of the model is the inclusion of other environmental factors, such as soil moisture and drought stress. In the dynamics model, the effect of temperature on growth stages was considered. However, the inclusion of other factors may allow for improved fitting and simultaneous parameter estimation of multiple environments. For example, the low heritability in 2018 of the growth parameters was due to severe heat stress in the summer of 2018, which made the growth slower than usual years. As a result, the sigmoid pattern in growth was truncated at the end of the cultivation period (Figure 4). Other environmental factors will allow simultaneous parameter estimation in other environments, leading to stability in the estimated parameters.

### Prediction of Growth Curves

In CV1, the accuracy was close between GP and TGP (Figure 7). This result suggests that the dynamics model used in TGP could extract sufficient genetic variations from phenotypic variations in the GF dynamics pattern to achieve the same predictive accuracy as the GP.

Models with multivariate GP yielded better accuracy than those with univariate GP; the accuracies of MGP and TMGP were higher than those of GP and TGP, respectively. High correlations among variates, a typical property of dynamic data, suggest that multivariate GP improves the prediction accuracy because MTG and TMGP can leverage the among-characteristics correlation. In the following, we focus on the comparison between MGP and TMGP.

In 2018, the accuracy of TMGP was lower than that of MGP in CV1 and CV2 (Figure 7) because of the low heritability of the LAI dynamics model parameters. However, the accuracy of TMGP was higher than that of MGP in 2019 for CV2. TMGP was better than MGP because of the higher heritability of growth parameters than GF in 2018. The extraction of genetic variance in growth patterns in 2019 was successful as LAI_amp_ in 2019-C and T*_*g*_* in 2019-D, leading to improved prediction accuracy.

In CV3, the prediction accuracies of TGPG and TMGPG outperformed the other models (Figure 7). The higher prediction accuracy compared to MGP indicates that the former growth period’s data could be effectively included in the model by specifying the growth curve’s shape through the dynamics model. In most cases, the correlation coefficients between the predicted values of TMGPG and the genotypic values exceeded 0.6, indicating that TMGPG is robust to changes in the environment. The similar prediction accuracy of MGP and TMGPG in 2017-D may be due to the lack of change in the GF in the second half of this environment’s growth period. This approach to future prediction through dynamic models has potential applications for selection in early growth stages in crop breeding.

In this study, the dynamics model and GP/MGP were used separately in TGP/TMGP, but they could be integrated into one hierarchical model. Several reports have shown the effectiveness of hierarchical models in the analysis of dynamic traits (Onogi et al., 2019), quantitative trait loci analysis (Ma et al., 2002), and genome-wide association studies (Das et al., 2011; Crispim et al., 2015). The joint analysis is expected to make parameter estimation more robust. In this study, although two steps are required to estimate the growth parameters, joint estimation may simplify the estimation process further.

### Growth Analysis on Remote Sensing Data for Plant Breeding

Applying the dynamics model to crops monitored with UAVs allows us to capture the genetic variation in growth patterns. The combination of the dynamics model and genetic analysis was shown to be an efficient framework for analyzing our field experiments. It was able to predict future GF dynamics from observations covering only the early growth stages. This could contribute to reducing the cost of the field trials. This study suggests that data monitoring the experiment with UAVs and analyzing them using dynamics models and mixed models will benefit crop breeding.

Although this study applied the growth model that considers both growth and senescence to soybean GF, characteristics of growth curves vary depending on species, trait, or situation of observation. For example, a logistic curve that consider only growth was used for modeling stem diameter of forest tree (Ma et al., 2002) and power function was used for modeling leaf age of rice (Wu et al., 2002). Crop models that consider physiological mechanisms such as photosynthesis may be applied to consider effects of diverse environmental factors on dynamic traits. Even in such cases, the proposed framework is flexible enough to be applied. In particular, the future prediction of growth curves (CV3) is a characteristic method of this framework and is expected to be applied to various traits.

A random regression model is also known as a regression method of dynamic data with a mixed model structure, which was used in the GP of dynamic traits (Sun et al., 2017; Campbell et al., 2018). The strength of random regression lies in its simple formation, but it cannot incorporate the growth curve structure like the dynamics model in exchange. Our prediction framework attempts to improve the accuracy of future predictions by considering the features of growth curves in the modeling.

In the near future, UAV-RS is expected to play an active role in plant breeding and provide growth trajectory data from multiple breeding programs. It will be possible for breeders and researchers to focus on new genotypes to select and develop new varieties suitable for the target environment. The integrated use of dynamic models and GP will be a useful method to effectively link growth process data with marker genotype data to improve genetic gain for genomic selection.

## Data Availability Statement

The datasets of canopy area, daily temperature, and growth parameters can be found in the GitHub at https://github.com/YT100100/ReferenceData_2021_Frontires.

## Author Contributions

MT, MN, HisT, AK, and HI were involved in funding acquisition. AK prepared 198 accessions of soybean genetic resources. YT, GS, YO, YY, HirT, HidT, MT, HisT, AK, MN, TF, and HI conducted the field experiments. YT, GS, and HI conducted UAV remote sensing. HK-K prepared the genome-wide marker genotype data. YT, RL-L, and FB developed a dynamic model of the GF. YT analyzed the data and wrote the manuscript. HI supervised the study and edited the manuscript. All authors have reviewed and approved the final manuscript.

## Conflict of Interest

The authors declare that the research was conducted in the absence of any commercial or financial relationships that could be construed as a potential conflict of interest.

## Publisher’s Note

All claims expressed in this article are solely those of the authors and do not necessarily represent those of their affiliated organizations, or those of the publisher, the editors and the reviewers. Any product that may be evaluated in this article, or claim that may be made by its manufacturer, is not guaranteed or endorsed by the publisher.

## Footnotes

1
https://www.gene.affrc.go.jp/index_en.php

2
https://www.python.org

3
https://www.hiphen-plant.com
