# Supplementary material for: Genomic Prediction of Green Fraction Dynamics in Soybean Using Unmanned Aerial Vehicles Observations
Source: Front Plant Sci. 2022 Mar 16;13:828864. doi: 10.3389/fpls.2022.828864 (PMC8966771; doi:10.3389/fpls.2022.828864)
Supplement: Supplementary file 1 [file Data_Sheet_1.docx]

Supplementary Material

# Supplementary Figures and Tables

| Year | Sowing date | Thinning date | UAV-RS |
| --- | --- | --- | --- |
| 2017 | 4 Jul. | 24 Jul. | 26 Jul. – 21 Sep. |
| 2018 | 3 Jul. | 20 Jul. | 22 Jul. – 28 Aug. |
| 2019 | 10 Jul. | 24 Jul. | 25 Jul. – 7 Sep. |

**Supplementary Table 1.** Sowing date, thinning date, and period of UAV-RS of the field experiment.


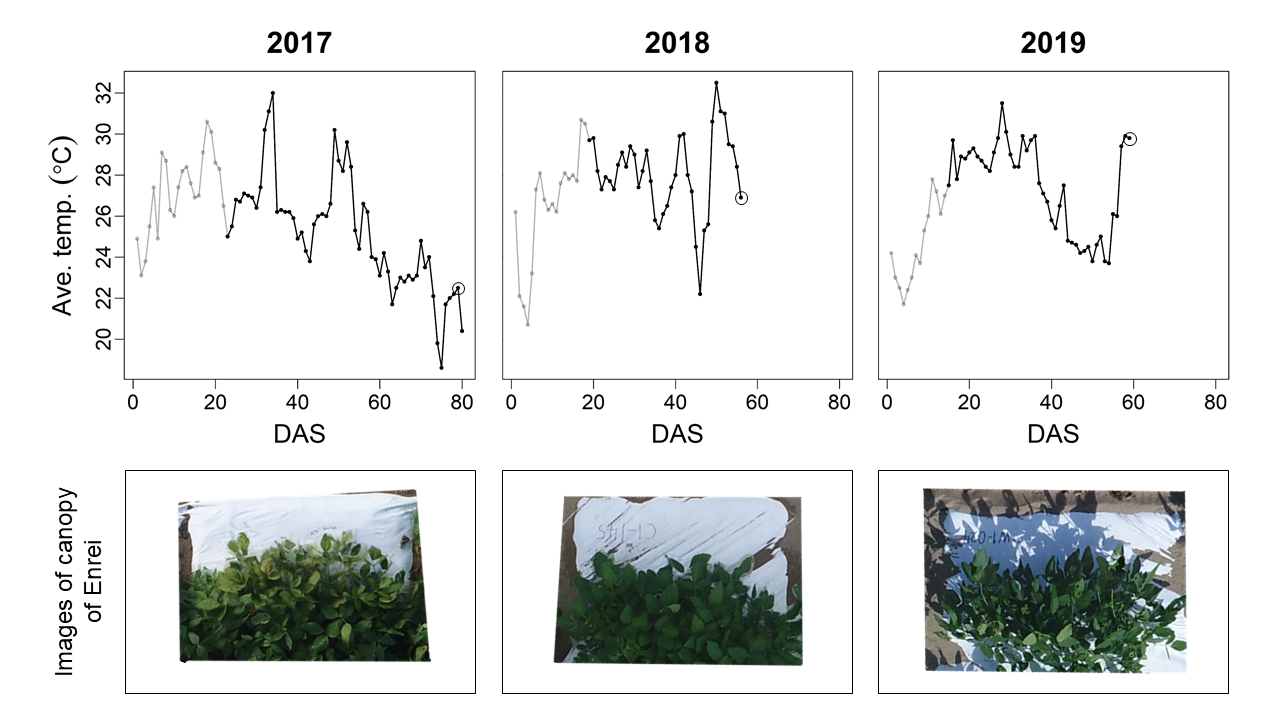


**Supplementary Figure 1.** (top) Change of daily mean temperature. Days after sowing (DAS) was used as the x-axis. Data taken before UAV-RS started is colored gray. Circles indicate the dates when the images of the canopy shown at bottom of this figure were taken. (bottom) Images of canopy obtained with UAV-RS on the final day in each year. A Japanese cultivar ‘Enrei’ in treatment C was chosen as an example.

**
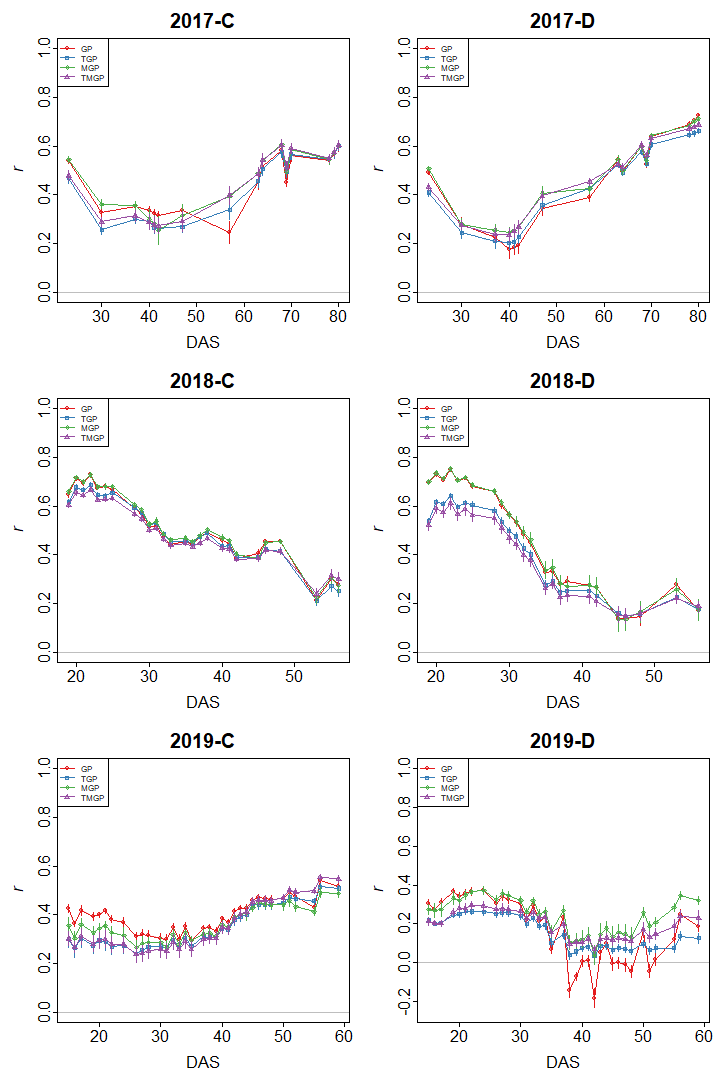
**

**Supplementary Figure 2.** The prediction accuracy of the canopy area using four models in CV1. The number of days after sowing (DAS) was used as the x-axis, whereas the correlation coefficients (*r*) between observed and predicted values yielded were plotted as the y-axis. The mean values of correlations yielded from repeated cross-validation were plotted as dots, and the range of ±1 standard deviations was expressed as vertical bars added to each dot. Red, blue, green, and purple lines correspond to the accuracy of GP, TGP, MGP, and TMGP, respectively.

**
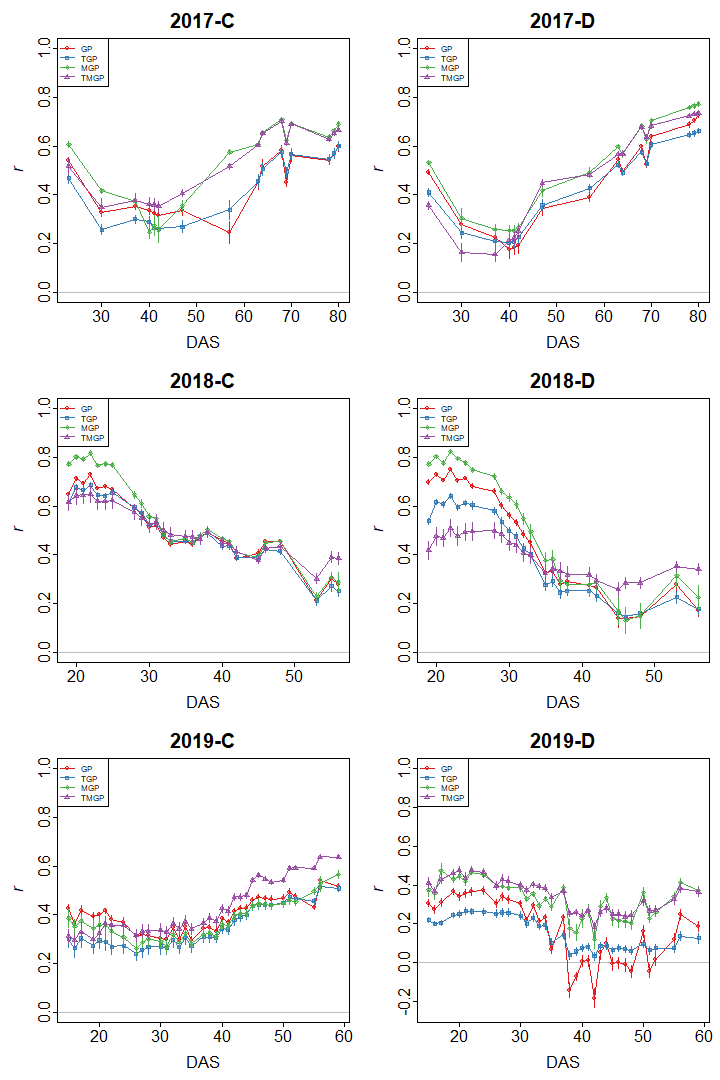
**

**Supplementary Figure 3.** The prediction accuracy of the canopy area using four models in CV2. The number of days after sowing (DAS) was used as the x-axis, whereas the correlation coefficients (*r*) between observed and predicted values yielded were plotted as the y-axis. The mean values of correlations yielded from repeated cross-validation were plotted as dots, and the range of ±1 standard deviations was expressed as vertical bars added to each dot. Red, blue, green, and purple lines correspond to the accuracy of GP, TGP, MGP, and TMGP, respectively.


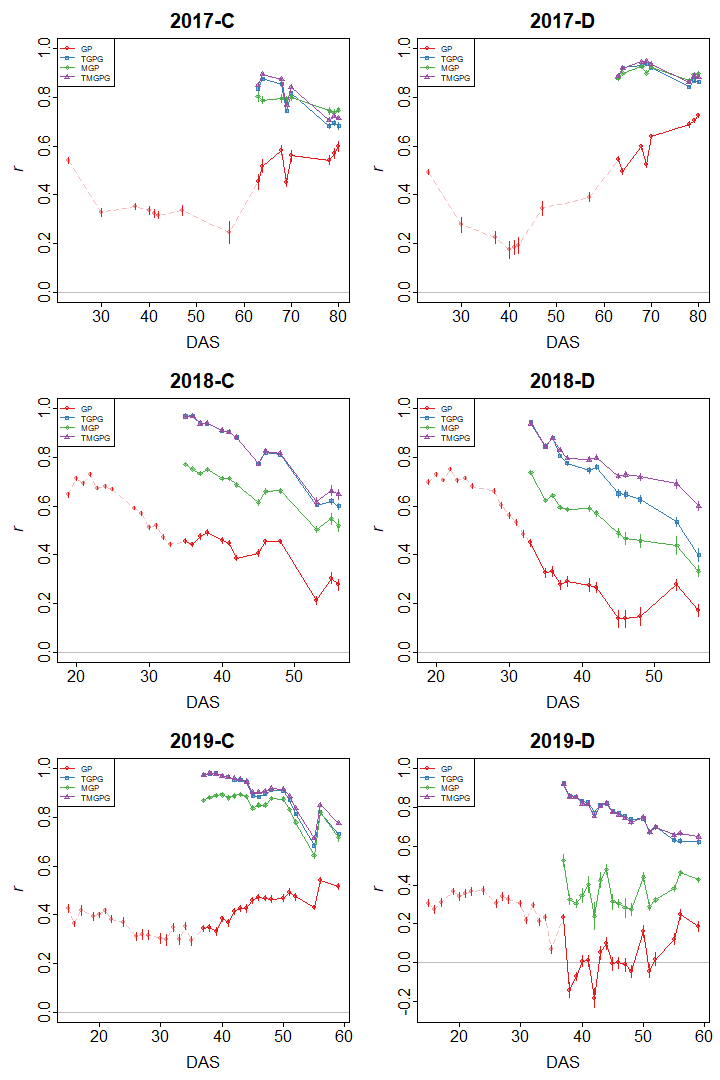


**Supplementary Figure 4.** The prediction accuracy of the canopy area using four models in CV3. The number of days after sowing (DAS) was used as the x-axis, whereas the correlation coefficients (*r*) between observed and predicted values yielded were plotted as the y-axis. The mean values of correlations yielded from repeated cross-validation were plotted as dots, and the range of ±1 standard deviations was expressed as vertical bars added to each dot. Red, blue, green, and purple lines correspond to the accuracy of GP, TGPG, MGP, and TMGPG, respectively. The accuracy in the former half of the growth period of GP is shown in fine points.
